# Newborn Screening for Cystic Fibrosis in Russia: A Catalyst for Improved Care

**DOI:** 10.3390/ijns6020034

**Published:** 2020-04-14

**Authors:** Victoria Sherman, Elena Kondratyeva, Nataliya Kashirskaya, Anna Voronkova, Victoria Nikonova, Elena Zhekaite, Sergey Kutsev

**Affiliations:** Research Centre for Medical Genetics, 115522 Moscow, Russia; elenafpk@mail.ru (E.K.); kashirskayanj@mail.ru (N.K.); voronkova111@yandex.ru (A.V.); nikonovavs@mail.ru (V.N.); elena_zhekayte@mail.ru (E.Z.); kutsev@mail.ru (S.K.)

**Keywords:** cystic fibrosis, newborn screening, IRT, sweat test

## Abstract

In order to assess the effectiveness of the detection of cystic fibrosis (CF) patients by screening compared with diagnoses based on clinical manifestations, the data of the National CF Patient Registry (NCFPR) from the year 2012 (group I: children aged 6–9 years, diagnosed prior to the start of screening) were compared with the data in the NCFPR from the year 2015 (group II: children 6–9 years after the start of screening) for CF patients from the Moscow region. Homozygotes for c.1521_1523delCTT (F508del) were separately compared in both groups. The average diagnosis age, genotype, body mass index, spirometry data, pulmonary infection, medications, and presence of complications were analyzed. This study demonstrated that in the c.1521_1523delCTT (F508del) homozygote group, the patients diagnosed by screening had significant advantages over the patients born before the start of newborn screening in the diagnosis age, the number of patients with chronic *Pseudomonas aeruginosa* infection, the pulmonary function, and the growth in the percentiles. Newborn screening (NBS) detects nearly twice as many CF patients as the diagnostics based on clinical symptoms during the same time period. Importantly, patients will benefit from the early diagnosis of the disease and the early start of therapy.

## 1. Introduction

In most European countries, newborn screening (NBS) is the main method for identifying patients with cystic fibrosis. Cystic fibrosis (CF) is monogenic disease with an autosomal recessive type of inheritance, and is common among Europeans and residents of North America. As a result of pathogenic variants of the *CFTR* gene (cystic fibrosis transmembrane conductance regulator), the protein that acts as the chloride channel in the body does not function, or its function is disordered in such a way that the viscosity of all of the secretions of the exocrine glands increase significantly, primarily secretions of bronchial mucus, the paranasal sinuses, the pancreas, and the liver. Hence, there is diversity in the disease’s clinical presentation, thus defining the polyorganic nature of the disease [1,2]. The degree of protein function disorder is determined by the type of gene pathogenic variant. More than 2000 *CFTR* pathogenic variants that might result in a disease phenotype have been identified; these pathogenic variants can be grouped into five or even six classes based on their effects on *CFTR* protein production, trafficking, function, and stability. In a five-class system, the pathogenic variants of the I–III classes are associated with classical CF and are considered “severe”, as they lead to significant disorders of the exocrine function of the pancreas. The pathogenic variants of the IV and V classes, in which the function of the chloride channel is partially preserved, are classified as “mild”. A VI class has subsequently been proposed, which is also associated with severe functional and phenotypic consequences [3,4,5]. Before the era of newborn screening, the diagnosis of CF was primarily based on clinical symptoms, such as the combination of respiratory and gastro-intestinal syndromes together with a delay in physical development. Thanks to the introduction of newborn screening, the diagnosis of CF can and should be established in the first months of life [2,6,7,8]. The detection of CF at the preclinical stage is the main goal of the newborn screening program, as it allows for the prescription of adequate basic therapy after an integrated assessment of the child’s condition, thereby preventing or delaying the development of complications.

Since June 2006, in several regions of the Russian Federation (RF), including the Moscow region, the list of congenital diseases for newborn screening has been expanded. Since January 2007, a massive screening of newborns for phenylketonuria, congenital hypothyroidism, galactosemia, congenital adrenal hyperplasia, and cystic fibrosis has been conducted throughout the Russian Federation.

Because of the existence of the registries of CF patients in a number of countries, as well as the annual reports of the European and North American registries, it is possible to assess what significant contributions newborn screening makes to the diagnosis of this severe disease. According to the National CF Patient Registry (NCFPR), in 2016, 44.7% of patients reported in the register were diagnosed based on the results of newborn screening [9]. Annually, about 200 new CF patients are diagnosed in Russia, with more than 70% diagnosed through newborn screening. According to the RF Ministry of Health, during the >10 years of the national newborn CF screening program in the RF (from 2007 to 2017 inclusive), more than 18 million newborns were screened, and CF was diagnosed in more than 1800 children. The average frequency over these 10 years was 1:9689 newborns (minimum frequency 1:8571 in 2011 and maximum frequency 1:10,498 in 2010).

The implementation of newborn screening for CF not only changed the approach to diagnosing the disease, but also changed the clinical characteristics of patients, especially children.

Many studies have been carried out to assess the effectiveness of newborn screening. The results of these studies suggest that newborn screening for cystic fibrosis positively affects the survival of patients [10,11], prevents the development of severe complications [12,13], improves physical development [14,15,16,17,18,19,20], improves lung function [15,18,21,22], and reduces the need for hospitalization [15]. In addition, the screening of newborns is a rare type of intervention carried out by health authorities, which is of benefit not only to patients, but also to all of society in economic terms [23,24].

The objective of our study was a comparative assessment of the clinical state and analysis of a number of indicators in groups of 6–9-year-old patients with CF from the Moscow region, who were diagnosed before and after the start of newborn screening.

## 2. Materials and Methods

The diagnosis of CF was proven by typical pulmonary or gastrointestinal symptoms or positive neonatal screening, or the diagnosis of CF in a sibling, as well as at least one of the following: two positive chloride sweat tests, or the identification of two CFTR pathologic variants in trans [2,6].

**Figure 1:**
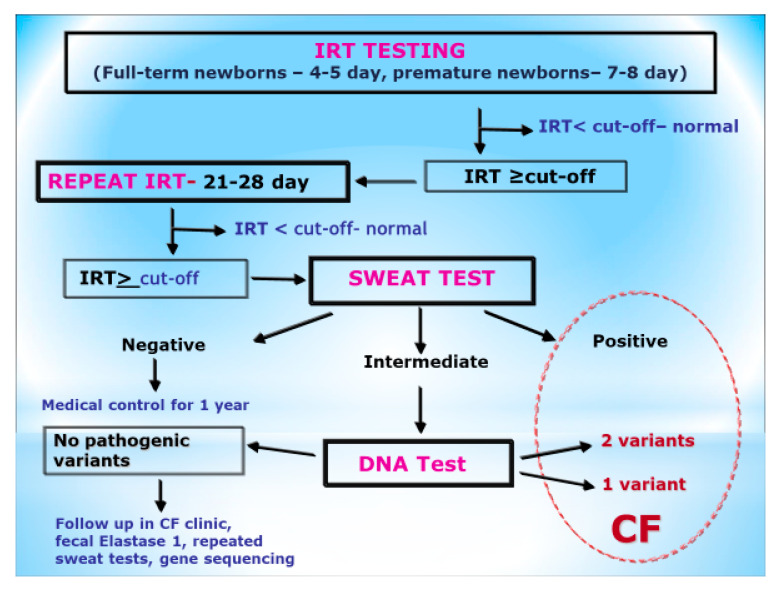
The algorithm of newborn screening for cystic fibrosis in Russia.

The screening protocol for CF in Russia comprises the following two stages: a double determination of immunoreactive trypsinogen (IRT) in the blood of newborns, and a sweat test in infants with an IRT concentration above the threshold during the second stage [6,25,26,27] (Figure 1).

IRT measurement was undertaken using the Delfia™ technique (heterogeneous time resolved fluorometric assay).

The cut-off for IRT at the first stage changed during those years from the 98.5th centile to the 98th centile (70 ng/mL to 65 ng/mL), because in the first year of the program, some children were missed. Infants with a raised second IRT measurement (IRT at the second stage greater than 40 ng/mL) at day 21–28 were referred for sweat testing at the local Neonatal Screening Laboratory or Regional CF Center. Infants with a normal sweat test were monitored by their local doctor for one year. Infants with an equivocal sweat test result were offered DNA analysis for common CF-causing mutations. Infants with a positive sweat test were referred to CF Center.

Prior to neonatal screening in the Russian Federation, a sweat test was carried out, mainly using the classical Gibson–Cook method. This test was not available in many hospitals and was often performed with protocol violations, which in turn led to the under- and over-diagnoses of the disease. At the Russian Center of Cystic Fibrosis in the early 2000s, a study of these methods was carried out to determine sweat conductivity using the following apparatus: Macroduct, Sweat-Chek, and Nanoduct (Wescor, USA). The positive experiences in using these techniques over several years allowed for the personnel of the CF Center to recommend the Nanoduct system for newborn screening confirmation. The Ministry of Health of the Russian Federation provided Nanoduct systems to genetic centers and large children’s medical institutions in all regions of the Russian Federation. This rapid diagnostic method allows one to receive quick and accurate results, with little or no intervention in the life of the child [26,27,28,29,30,31]. Numerous studies in the literature, combined with many years of our own experience, show a good correlation between the determination of conductivity and the quantitative method of measuring the concentration of sweat chlorides [32,33,34,35].

The *CFTR* testing algorithm included several steps. The Wizard Genomic DNA Purification Kit (Promega, USA) was used for DNA extraction from the whole blood samples, where EDTA was used as an anticoagulant. Initially, we examined the 34 most common *CFTR* variants utilized for the diagnosis of CF within the multiethnic RF, which account for over 85% of all CF-causing variants [1]. In-house molecular genetic methods that have been previously described [2], including amplified fragment length (AFLP) and restriction fragment length (RFLP) polymorphism techniques, were utilized to detect insertion/deletion variants and nucleotide substitutions, respectively. For the cases when one or both *CFTR* pathogenic variants remained unidentified, we carried out direct Sanger DNA sequencing of the entire *CFTR* coding region, including adjacent splice sites and the 3′-untranslated *CFTR* region [2].

We analyzed the data of 131 CF patients from the NCFPR from 2012 (children aged 6–9 years, diagnosed prior to screening only by typical symptoms) and 2015 (children 6–9 years after the start of screening) for patients with CF living in the Moscow region. All of the patients were under dynamic supervision by the personnel of the scientific and clinical department of cystic fibrosis of the Research Centre for Medical Genetics (Moscow) from the moment of diagnosis.

All 131 patients were divided into two groups, namely: Group I—patients diagnosed prior to the start of the NBS program (45 persons); Group II—patients diagnosed by NBS (86 persons; Table 1).

In the studied groups, the following indices of NCFPR were analyzed and compared: sex, age of diagnosis, genotype, number of patients with normal levels of fecal pancreatic elastase 1 (>200 μg/g), the presence of *Staphylococcus aureus* and *Pseudomonas aeruginosa*, diabetes, liver disease, hemoptysis, osteoporosis, nasal polyposis, amyloidosis, respiratory function indices (forced vital capacity (FVC), forced expiratory volume in the first second (FEV_1_)), nutritional status (height, weight, and body mass index (BMI)), and medications (pancreatic enzyme therapy, mucolytics, antibacterial, and anti-inflammatory therapy). The NCFPR contains the same variables as in the European CF Society Patient Registry.

In Group I and Group II, we identified two subgroups of c.1521_1523delCTT (F508del) homozygotes, namely: subgroup IA (CF patients diagnosed prior to the CF NBS program), comprising 20 persons, and subgroup IIA (diagnosed by CF NBS), comprising 21 persons.

The data obtained were processed using the STATISTICA software package. As a measure for describing the original sample, the arithmetic mean (M) and standard deviation (SD) criteria were used, while an interpretation of findings (not having a normal distribution) was carried out using the median (Me) and the interquartile range (IQR). In order to compare the samples obtained according to the quantitative criterion, a Mann–Whitney U-test was used. Differences were considered statistically significant at *p* < 0.05.

## 3. Results

The studied groups varied significantly in their number of patients. Thus, in Group I (before screening), there were only 45 people, while in Group II (by screening), there were 86 children. This suggests that the NBS program can detect twice as many patients compared with the same period before the start of the screening program. Groups I and II did not vary in sex, as each group contained an approximately equal number of boys and girls (Table 1).

A significant difference was found between Groups I and II for the age of CF diagnosis. The mean age of diagnosis in Group I was 2.29 (±2.29) years, and the median was 1.17 (0.5–4.08) years. In Group II, the mean age of diagnosis was 0.66 (±1.13) years (*p* = 0.0001), and the median was 0.19 (0.11–0.48) years (Table 2).

Prior to the start of the NBS, the diagnosis was mainly determined by clinical symptoms. Therefore, the patients with “severe” pathogenic variants prevailed in Group I. In this group, the homozygous carriage of the most common “severe” pathogenic variant, c.1521_1523delCTT (F508del), was 44.4% (20 patients out of 45), and the allele frequency of c.1521_1523delCTT (F508del) was 62.2%.

In Group II, the genotype c.1521_1523delCTT (F508del)/c.1521_1523delCTT (F508del) was found in 24.4% of patients (21 of 86), and the allele frequency of c.1521_1523delCTT (F508del) was found in 40.7%. The median age of CF diagnosis among the c.1521_1523delCTT (F508del) homozygotes was also significantly lower in Group II (0.19 (0.11–0.35) years) compared with Group I (1 (0.41–4.08) years; Table 3).

No difference was found in the nutritional status (weight, height, and BMI) between Groups I and II. A fecal elastase 1 value of more than 200 μg/g of stool (normal range) was detected in four (8.9%) children in Group I and in 19 (22.1%) patients in Group II (Table 2). However, when we compared the indices of nutritional status in the subgroups of the c.1521_1523delCTT (F508del) homozygotes, we found significantly better growth in the subgroup IIA indices (57.4 (36.4–79.1) percentile) and subgroup IA (21.7 (11.9–52.7) percentile. No significant differences were obtained by weight and BMI (Table 3).

Special attention was paid to the examination of differences in the lung function indices, which are one of the most important characteristics for the course of the disease and its prognosis in CF patients. In general, patients diagnosed by the NBS program did not differ in their FVC and FEV_1_ from their peers identified according to their clinical symptoms (Table 2).

In contrast, a significant difference was demonstrated in the two subgroups of c.1521_1523delCTT (F508del) homozygotes. The mean FVC in subgroup IA was 80.0 ± 25.5%, while that in the subgroup IIA was 102.0 ± 11.2%. The average level of FEV_1_ in subgroup IA was 64.0 ± 31.1%, while that in subgroup IIA was 104.6 ± 11.9% (Table 3).

Based on the results of the evaluation and comparison of the microbiological status (the presence of the chronic colonization of *Staphylococcus aureus* and chronic or intermittent *Pseudomonas aeruginosa*), Group II (diagnosed by NBS) was significantly less likely to have a chronic *Pseudomonas aeruginosa* infection (37.8% were infected with *Pseudomonas aeruginosa* in Group I versus 12.8% in Group II; Table 2). Those with c.1521_1523delCTT (F508del) homozygotes, diagnosed by NBS, experienced chronic *Pseudomonas aeruginosa* infection significantly less frequently (9.5% in subgroup IIA and 55% of patients in subgroup IA; Table 3)

A statistically significant decrease in the volume of intravenous and inhalation antibiotic therapy was observed in Group II, as well as a decrease in the number of patients receiving bronchodilators (Table 4).

This can be explained by the decrease in the number of patients with *Pseudomonas aeruginosa* infection who, consequently, experienced fewer exacerbations of the chronic bronchopulmonary process. Patients from Group II were less often prescribed systemic steroids, which may indicate a lower severity of the disease (Table 4). Those with c.1521_1523delCTT (F508del) homozygotes from subgroup IIA required inhalations of antibiotics and bronchodilators much less frequently than the patients from subgroup IA (Table 3).

Our analysis did not reveal any significant difference in the frequency of nasal polyposis and liver damage between Groups I and II, including those with c.1521_1523delCTT (F508del) homozygotes.

## 4. Discussion

Presently, NBS is the most important and unique method for detecting the maximum number of CF patients at an early preclinical stage. Our study has shown that the NBS makes it possible to identify almost two times more patients than diagnosis that relies on clinical signs over the same period of time. At the same time, it is possible to diagnose CF in patients with “mild” pathogenic variants, for whom the course of CF can be easier because of the preserved function of their pancreas. The early delivery of complex therapy will help to slow down or even prevent the development of complications in these patients.

In Russia, the effectiveness of newborn screening for CF has previously been evaluated [20]. The National CF NBS program has resulted in an earlier detection of infants with CF and a clearer picture of the incidence of this condition in the population. The NBS has dramatically driven an improvement in the provision of CF care across Russia. In 2012, a study was performed on a group of Moscow CF patients of an early age (<3 years old). This study showed that, when compared with children diagnosed at an older age, the condition of patients diagnosed in the first months of life by the NBS was better in terms of their physical development, bronchopulmonary system, microbiological status, number of exacerbations of gastrointestinal and respiratory syndromes, and morbidity [20]. The present study was performed on a group of older CF children (6–9 years of age). A number of advantages in the Group II patients diagnosed according to the NBS have been demonstrated.

A comparison of homozygotes for c.1521_1523delCTT (F508del) from subgroups IA and IIA showed even more significant differences. Thus, the patients from subgroup IIA showed higher indices of physical development (height) and lung function (FVC, FEV_1_) compared with their peers in subgroup IA, as well as a lower age of diagnosis, a lower frequency of chronic *Pseudomonas* infection, and a lesser need for antibacterial therapy.

Chronic lower respiratory tract infection is a key symptom in CF patients [1,2], and is a leading factor in determining the severity and prognosis of the disease. When studying the microflora of the lower respiratory tract in different age groups of CF children, researchers from different countries found that the main pathogens of lung infection in CF patients are *Pseudomonas aeruginosa*, *Staphylococcus aureus*, and *Haemophilus influenzae.* In the first years of life, for patients with CF, *Staphylococcus aureus* dominates; then, *Pseudomonas aeruginosa* becomes the main pathogen [26]. Chronic *Pseudomonas aeruginosa* infection is clearly associated with worse outcomes, including survival, lung function, numbers of pulmonary exacerbations, and nutritional status [35].

Concerning the important physical development indicators of growth, short stature (or stunting—an indicator of chronic malnutrition) is a persistent problem in children with CF [36] and is also a prognostic factor [37]. Despite an improved nutritional status and median survival age, many individuals with CF still fail to reach the average height for their age [38] and/or their genetic potential for height. Children who have their cystic fibrosis diagnosed early through newborn screening present better nutritional status and growth, with greater height percentiles during the first decade of life compared with those who were diagnosed by traditional methods after presenting signs/symptoms and then received similar standard nutritional therapies. Not-screened CF patients also experienced catch-up growth after the declined height status at CF diagnosis, but never reached the same level as the screened patients did. The Wisconsin RCT project demonstrated that the early diagnosis of CF within weeks of birth provides a great opportunity to prevent detrimental nutrition and growth retardation in early infancy. In conjunction with appropriate nutritional therapy, these early growth benefits of NBS sustain over the long-term, through puberty, and can lead to greater adult height [38]. It should also be noted that stunting is an important social and psychological factor that determines the adaptation of a person in society.

In both groups of patients, there were practically no cases of complications of CF (e.g., cystic fibrosis-associated diabetes mellitus, pulmonary hemorrhage, pneumothorax, amyloidosis, and osteoporosis; Table 2), which can easily be explained by the young ages of the patients, as most of these complications develop, as a rule, in adolescents and adults [9].

## 5. Conclusions

The main conclusion of the present study is a confirmation of the importance of mass newborn screening to identify the maximum number of patients with CF at a very early preclinical age. In addition, it was shown that the NBS offers a number of advantages not only for those in the early period of life, but also for children from older age groups. The method of medical care for CF newborns identified by NBS (as used by the personnel of the Russian Center of Cystic Fibrosis (Research Centre for Medical Genetics)) has also shown its effectiveness. Thus, the early detection of patients and outpatient monitoring systems with measures to prevent cross-infection, alongside regular monitoring of the microbiological status, made it possible to reduce the incidence of chronic *Pseudomonas aeruginosa* infections in the NBS group, and, as a consequence, reduced the need for antibacterial therapy.

## Figures and Tables

**Table 1:** The characteristics of the patient groups.

Group of Patients 6–9 Years	Group I (*n* = 45) (before NBS)	Group II (*n* = 86) (after NBS)
Female	25 (55.6%)	44 (51.2%)
Male	20 (44.4%)	42 (48.8%)
Age (years), mean ± SD	8.2 ± 1.0	7.7 ± 1.1
Patients c.1521_1523delCTT (F508del)/c.1521_1523delCTT (F508del)	20 (Group IA)	21 (Group IIA)

**Table 2:** Clinical characteristic of the cystic fibrosis patients in Groups I and II.

Group of Patients 6–9 Years (*n*)	Group I (before Screening) (45)	Group II (after Screening) (86)	*p*
Age at diagnosis (years), median (IQR)	1.17 (0.50–4.08)	0.19 (0.11–0.48)	0.0001
Fecal elastase 1 >200 µg/g	4 (8.9%)	19 (22.1%)	(*)
Chronic *Staphylococcus aureus* infection	38 (84.4%)	69 (80.2%)	0.5252
Chronic *Pseudomonas aeruginosa* infection	17 (37.8%)	11 (12.8%)	0.0026
Intermittent *Pseudomonas aeruginosa* infection	6 (13.3%)	12 (13.9%)	0.6662
Diabetes	1 (2.2%)	0	0.1797
Liver damage	3 (6.7%)	7 (8.1%)	0.6954
Synonasal polyposis	12 (26.7%)	15 (17.4%)	0.2579
FVC (%), median (IQR)	86.0 (74.0–98.0)	88.0 (79.0–100.0)	0.4311
FEV_1_ (%), median (IQR)	87.0 (65.0–93.0)	89.0 (78.0–108.0)	0.2667
Height (percentile), median (IQR)	49.8 (17.8–74.0)	52.8 (30.7–79.1)	0.2377
Weight (percentile), median (IQR)	24.0 (20.0–29.0)	23.5 (21.0–25.9)	0.3952
BMI (percentile), median (IQR)	43.2 (11.5–65.0)	37.3 (17.3–68.1)	0.6749

* The statistical reliability was not calculated because of the insufficient size of the groups.

**Table 3:** Characteristics of the homozygotes c.1521_1523delCTT (F508del) in subgroups IA and IIA.

	Group IA (*n* = 20) (before NBS)	Group IIA (*n* = 21) (after NBS)	*p*
Age at diagnosis, years, median (IQR)	1 (0.41–4.08)	0.19 (0.11–0.35)	0.0017
Chronic *Pseudomonas aeruginosa* infection	11 (55.0%)	2 (9.5%)	0.0027
FEV_1_ (%), mean ± SD	64.0 (±31.1)	104.6 (±11.9)	0.0084
FVC (%), mean ± SD	80.0 (±25.5)	102.0 (±11.3)	0.0247
Height (percentile), median (IQR)	21.7 (11.9–52.7)	57.4 (36.4–79.1)	0.0182
Weight (percentile), median (IQR)	28.3 (6.9–56.0)	49.2 (21.8–77.1)	0.0565
BMI (percentile), median (IQR)	24.7 (11.5–59.2)	40.7 (23.0–73.3)	0.1964
Inhalation antibiotics	15 (75.0%)	6 (28.6%)	0.0142
Bronchodilators	18 (90.0%)	10 (47.6%)	0.0304

**Table 4:** Use of different treatment regimes and medicaments in the Groups I and II.

Group of Patients 6–9 years (*n*)	Group I (*n* = 45) (before NBS)	Group II (*n* = 86) (after NBS)	*p*
Inhaled hypertonic saline	17 (37.8%)	62 (72.1%)	0.0001
Inhaled antibiotics	26 (57.8%)	28 (32.6%)	0.0139
IV antibiotics	17 (37.8%)	5 (5.8%)	0.0001
Oral antibiotics	40 (88.9%)	67 (77.9%)	0.2693
Bronchodilators	35 (77.8%)	47 (54.6%)	0.0322
Inhaled corticosteroids	2 (4.4%)	4 (4.6%)	0.8895
Systemic corticosteroids	4 (8.9%)	1 (1.2%)	(*)
Pancreatic enzymes	44 (97.8%)	71 (82.6%)	0.0546

* The statistical reliability was not calculated due to the insufficient size of the groups.
